# Correcting fast irregular motion in PET: maximum-likelihood motion and activity (MLMA) reconstruction

**DOI:** 10.1186/s40658-026-00894-0

**Published:** 2026-05-26

**Authors:** Rodrigo José Santo, Ethan Waterink, Cornelis A.T. van den Berg, Thomas Olausson, Alessandro Sbrizzi, Hugo W.A.M. de Jong, Casper Beijst

**Affiliations:** 1 Department of Radiotherapy, Imaging & Oncology Division, UMC Utrecht, Utrecht, The Netherlands; 2 Department of Radiology & Nuclear Medicine, UMC Utrecht, Utrecht, The Netherlands

**Keywords:** Motion correction, Gateless motion-corrected PET, High-frequency motion-corrected PET, irregular non-rigid motion correction

## Abstract

**Purpose:**

Positron emission tomography (PET) imaging naturally suffers from motion blur due to long acquisitions. As such, motion-compensation provides a promising solution to improve image quality. Traditional methods for motion-correction often involve a combination of gating and data-binning, assuming that motion is periodic, to accumulate sufficient counts per motion-state frame. Irregular motion can be estimated but it requires complex motion-capture systems or elaborate data-driven algorithms, which are difficult to configure (physical or model setup) and hampered by the high noise of short timeframes. We propose a new method that alternatingly estimates and corrects for motion at high temporal frequency in PET imaging: Maximum-Likelihood Motion and Activity (MLMA) reconstruction. MLMA estimates both the time-series of deformation vector fields and the motion-corrected activity image for the whole acquisition. Together, this allows to visualize anatomical structures moving in time.

**Methods:**

The method exploits the high compressibility and spatial smoothness of motion through a cubic B-spline motion-model and through spatial regularization. MLMA was configured to 2 Hz resolution and applied on (A) the digital XCAT phantom, (B) acquisitions of a moving anthropomorphic torso phantom and (C) clinical patient data.

**Results:**

The results show that MLMA can accurately correct motion at high frequency (2 Hz), with subvoxel accuracy (up to 2.5 mm root-mean-square error (RMSE) on 4 mm isotropic voxels) and realistic breathing (amplitude range 14.7 mm and average period 4.5 s). This enables visually-noticeable improvements on image quality.

**Conclusion:**

The proposed MLMA reconstruction method resolves the motion encoded in very-short PET timeframes, irrespective of the very low counts and noise inherent to PET projection data.

**Supplementary Information:**

The online version contains supplementary material available at 10.1186/s40658-026-00894-0.

## Introduction

Positron emission tomography (PET) imaging is associated with acquisition times of numerous minutes, due to the low radiotracer doses used. Therefore, motion of the patient during the acquisition introduces blurring and consequently degrades diagnostic accuracy [1]. Typically, this originates from patient repositioning, head motion, respiratory motion and/or cardiac motion. In many applications, these effects are critical as is the case in pediatric PET, where anesthesia is typically used to immobilize children [2]. This is required as fear and anxiety lead to irregular aperiodic motion/rolling/sliding. Although effective, the cumulative exposure may contribute to deleterious neurodevelopmental effects [3], prompting the need for better motion management.

For periodic motion such as respiratory or cardiac motion, correction approaches often arrange the acquired data into gates/phases. Each gate approximately corresponds to the same motion state, defined by an external surrogate signal (respiratory belt and/or electrocardiogram (ECG)) or by retrospective data analysis (data-driven approaches) [1]. This allows to accumulate enough signal to reconstruct images of enough quality for each gate, from which motion is estimated via registration and used for a final motion-corrected reconstruction. Another approach is to incorporate motion in the PET signal model [4–8] for a joint estimation of activity and motion. These methods are intrinsically limited to the anatomical motion correlated to the gating signal.

For irregular aperiodic motion such as deep inhale/exhale and patient drift/repositioning, motion-correction is more challenging. Complex external motion-capture systems enable continuous tracking of the patient position, through cameras/lasers [9] or separate imaging modalities such as magnetic resonance imaging (MRI) [10]. Data-driven approaches estimate the motion from noisy reconstructions of short timeframes, through conventional image registration [11, 12] or deep-learning [13]. These methods are limited to affine motion models and require optimal configuration (physical setup or model parameters).

To overcome the limitations of current approaches, we propose to estimate motion directly from list-mode data. The rationale is that short timeframes represent different views of the same anatomical structures, modulated by physiological motion fields. By considering the anatomical structures as coherent in time, and by posing mild conditions on the nature of the modulating motion fields, the activity-motion reconstruction problem becomes well-posed. Therefore, integrating motion and activity in the unified reconstruction process allows to forgo gating and enables motion-correction at a high temporal resolution.

In this paper, we present a method to correct irregular and periodic motion in PET at a high temporal resolution: Maximum-Likelihood Motion and Activity (MLMA) reconstruction. It is built upon the MOTion from Undersampled Signals (MOTUS) framework, which has been successfully implemented for MRI [14, 15] and cone-beam computed tomography (CT) [16]. It was demonstrated on: (A) simulated data for a breathing XCAT phantom; (B) measured data of a moving anthropomorphic torso phantom and (C) patient data from a Biograph mCT (Siemens Healthineers) scanner.

## Materials and methods

**Fig. 1:**
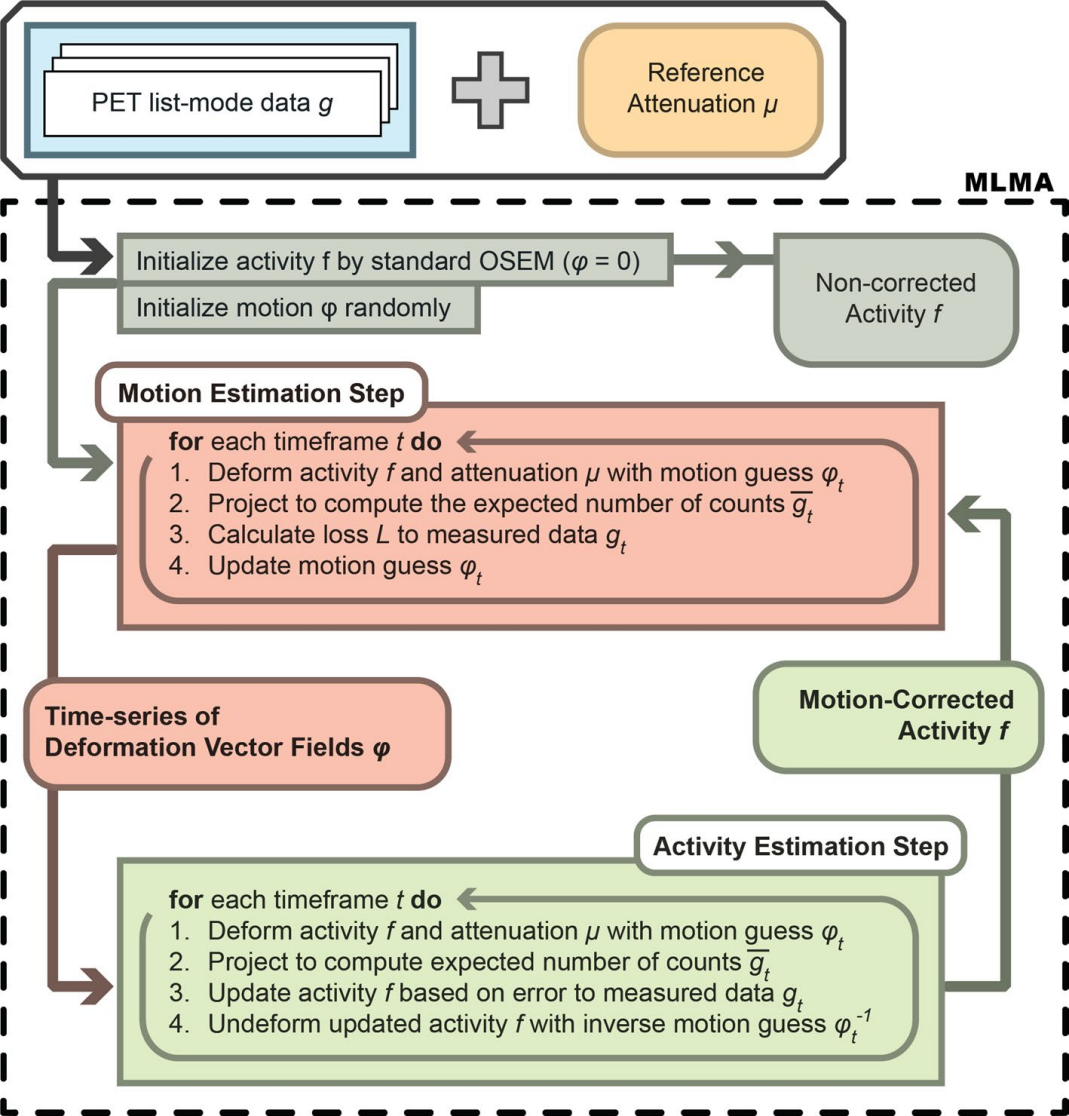
Schematic pseudocode of the proposed Maximum-Likelihood Motion and Activity (MLMA) reconstruction method

The Maximum-Likelihood Motion and Activity (MLMA) reconstruction method estimates the time-series of deformation vector fields $\:\boldsymbol{\varphi\:}$ and the motion-corrected activity *\:f*, from solely the PET list-mode data *\:g* and the reference attenuation map $\:\mu\:$ as illustrated in Fig. 1.

Following from the Poisson-distributed PET data, the MLMA reconstruction minimizes the negative log-likelihood objective function *\:-L* in an alternating way. For the activity estimation step, optimization is performed with regards to the activity *\:f*. The motion estimation step optimizes with regards to the motion fields $\:\boldsymbol{\varphi\:}$ for each timeframe *\:t* separately, using a regularized version $\:{\mathcal{L}}_{t}$ of the objective function, as described in the following section.

$$ \begin{aligned} \: - L\left( {f,\varphi \:,\mu \:} \right) & = - \sum {\:_{{t = 1}}^{{N_{t} }} } L_{t} \left( {f,\varphi \:_{t} ,\mu \:} \right),\:\:L_{t} \left( {f,\varphi \:_{t} ,\mu \:} \right) \\ & = \sum \: _{{i = 1}}^{{N_{b} }} \sum \: _{{j = 1}}^{{N_{v} }} \bar{g}_{{i,j}} \left( {f,\varphi \:_{t} ,\mu \:} \right) - {\mathrm{log}}\left( {\bar{g}_{{i,j}} \left( {f,\varphi \:_{t} ,\mu \:} \right)} \right)*g_{{t,i,j}} \:\:, \\ \end{aligned} $$

where *\:i* represents the line-of-response bin (LOR, total $\:{N}_{b}$), *\:j* is the time-of-flight bin (TOF, total $\:{N}_{v}$), *\:t* the timeframe number (total $\:{N}_{t}$), *\:g* is the list-mode data and $\:\stackrel{-}{g}$ the expected number of counts, obtained from deforming the activity map *\:f* and the attenuation map $\:\mu\:$ with the motion field $\:{\varphi\:}_{t}$ and projecting into the LOR-space. The expected number of counts $\:\stackrel{-}{g}$ takes into account the effects of normalization, randoms and scatters directly in the signal model. These contributions are estimated in the initialization step of MLMA, using the motion-blurred image to estimate the time-averaged correction factors.

### Motion estimation step

Motion estimation builds upon the rationale that short timeframes of PET data represent the same anatomical structures but in different views, modulated by physiological motion fields. As such, the motion estimation problem is made well-posed and solvable for the very low-counts short timeframes by assuming that the anatomical structures are coherent in time and the motion is spatially-smooth, which can be described by a highly-compressed motion model.

To highly-compress motion, the model was designed as a free-form deformation (FFD) parameterized by lower-dimension 3D cubic B-spline basis functions [17]. Spatial-smoothness was enforced by the Laplace operator, added to the objective function. Temporal-smoothness (deviation between consecutive timeframes) and compressibility (determinant of Jacobian) were also regularized. These terms are weighted and added to the negative log-likelihood for each timeframe, to compose the regularized objective function $\:{\mathcal{L}}_{t}$.The motion model is derived in the Supplemental Materials.

Motion estimation was implemented in *Python*, through the *PyTorch* [18] and the *ParallelProj* [19] libraries. To enable GPU acceleration, the projector of the motion estimation step was implemented without TOF.

### Activity estimation step

The activity estimation step used the CASToR [20] framework, with the ordered-subset expectation-maximization (OSEM) algorithm [21]. Deformable motion-correction was implemented through CASToR’s image transformation template module for involuntary motion, using the “deform and project” approach [22].

To overcome artifacts from the mismatch of the CT-based attenuation map with the motion-corrected PET images, the deformation vector fields were rewritten in terms of a reference frame after the first alternation. Consequently, the reconstructed motion-corrected activity image corresponds to the same position as the attenuation map. The reference frame was selected based on the alignment between the reference attenuation map and the deformed activity images by visual inspection.

### Experiments

MLMA was evaluated in three experiments, as in Table 1.

**Table 1:** Summary of the experiments to evaluate the Maximum-Likelihood Motion and Activity (MLMA) reconstruction method

Experiment	Dataset	Object	Motion type
**A**	Simulated	XCAT phantom	Free-breathing
**B**	Moving Platform	Anthropomorphic torso phantom	Sinusoidal Breathing-like Random
**C**	Clinical patient	Patient	Free-breathing

#### A XCAT phantom simulations

The XCAT digital phantom was used to simulate human-like respiratory motion. A 3-minute acquisition was simulated with randomly varying breathing amplitude (1–4 cm) and period (4–7 s), at a temporal resolution of 500 ms. A hot lesion was placed in the liver. Additionally, a static 3-minute acquisition was simulated for a randomly-selected frame of the moving acquisition. The corresponding static attenuation map was also used for reconstruction of the moving acquisition.

The resulting XCAT maps were projected into list-mode data using CASToR, for the geometry of the Biograph mCT. Poisson noise was applied, for clinically-consistent counts (10^6^ true counts per minute). Data was also reconstructed with gating, based on the ground-truth motion of the lesion. Reconstruction was performed using 6 gates defined by amplitude, followed by registration to the gate that better aligned visually with the attenuation map. The final motion-corrected image was obtained by summing the registered gates.

#### B Moving platform phantom acquisitions

The Data-Spectrum anthropomorphic torso phantom [23] was scanned on a moving platform at a Biograph mCT scanner. Different motions were applied in the cranio-caudal direction: sinusoidal, breathing-like and random as in Fig. 5. The maximum amplitude was configured to 3 cm and duration to 3 min. Additionally, a static 3-minute acquisition was performed with the phantom centered in the scanner field-of-view (FOV). The corresponding static attenuation map was also used for reconstruction of the moving acquisitions.

The phantom was filled with 19MBq of [^18^F]Fluorodeoxyglucose (FDG), at a ratio of 1:2.1 for liver-myocardium and 1:4.9 for background-myocardium. Data was acquired for 3 min in list-mode using the standard energy window for the moving experiments and the static acquisition. The clinical corrections for scatters and randoms estimated by the scanner were applied.

The programmed motion of the phantom was also used as a gating signal. Reconstruction was performed 6 gates defined by amplitude for each acquisition, followed by registration to the gate that better aligned visually with the attenuation map. The final motion-corrected image was obtained by summing the registered gates.

#### C Clinical patient data

MLMA was applied to clinical data of one patient, acquired in free-breathing at a Biograph mCT scanner. The patient was injected with [^18^F]Flurpiridaz (Flyrcado^®^, GE Healthcare) and a single bed-position covering the heart and liver-lung interface was used for a 3-minute reconstruction, using the clinical list-mode protocol. The corrections for scatters and randoms estimated by the scanner were used.

The clinical data was also reconstructed with gating, following a data-driven approach. The gating signal was obtained with the center of distribution (COD) method [24], to split the list-mode data into 6 gates based on amplitude. Reconstruction was performed for each gate, followed by registration of the activity images to the gate that better aligned visually with the attenuation map. The final motion-corrected image was obtained by summing the registered gates.

This study does not fall under the scope of the Dutch Medical Research Involving Human Subjects Act (WMO), therefore it does not require approval from an accredited medical ethics committee in the Netherlands. However, in our institution, an independent quality check has been carried out to ensure compliance with legislation and regulations (regarding informed-consent procedure, data management, privacy aspects and legal aspects).

### Performance evaluation

To evaluate motion, the estimated deformation vector fields were compared with the ground-truth, using the root-mean-square error (RMSE). For experiment (A) on the XCAT phantom, the motion was obtained by tracking the centroid of the lesion in the reference maps. For experiment (B) on the torso phantom, it was obtained from averaging the deformation vector fields on a region-of-interest (ROI) around the heart compartment. On the clinical patient data of experiment C), the motion profile was obtained from a 8 cm^3^ volume centered at the liver-lung interface.

For image quality, the comparison was performed both visually and through line profiles, against non-corrected (OSEM) images to evaluate relative improvements. For experiments A) and B), comparison was also performed against static references and gated motion-corrected images with quantitative metrics, such as peak signal to noise ratio (PSNR), Structural Similarity Index Measure (SSIM) and Variance. For the clinical patient data of experiment C), the results were compared to a gated motion-corrected image.

### Parameter settings

MLMA was configured for a temporal resolution of 2 Hz on an isotropic voxel size of 4 mm, for a volume of dimensions 128 × 128 × 54 voxels. A Gaussian filter was applied after activity estimation, with an isotropic kernel of 5 mm FWHM. Motion estimation used the NAdam optimizer [25]. MLMA was performed for 5 alternations, using 10 iterations for the motion update step and 3 iterations with 21 subsets for the activity estimation step. Reconstructions were performed in a desktop computer with a 32-core 3.6 GHz Ryzen Threadripper CPU, 256 GB of RAM and a Nvidia Quadro P6000 GPU. A description of the parameters is provided in the Supplemental Materials.

## Results

The reconstruction time of MLMA was the same for all experiments, clocking at 9 h. Per alternation, the motion estimation step took 180 min and the activity estimation step took 90 min. Reconstruction time for the gated-reconstruction was 15 min, including the overhead to sort the gates. For the clinical patient data, an additional 20 min were required to obtain the gating signal from the list-mode data.

### A XCAT phantom simulations

**Fig. 2:**
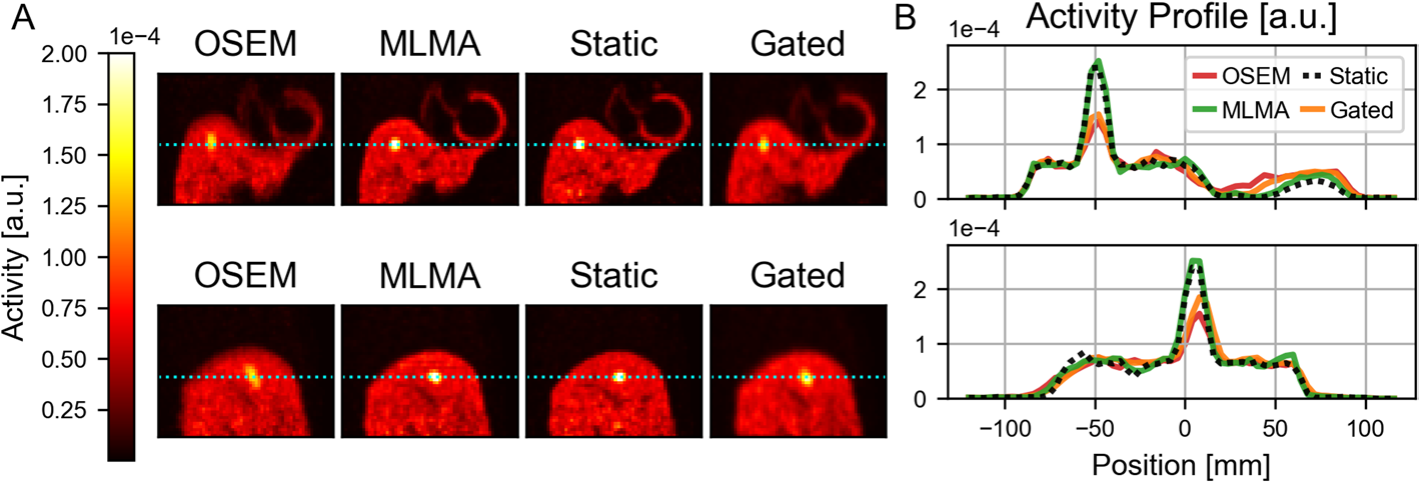
PET activity images (**A**) and line profiles (**B**) for the proposed MLMA, for the standard reconstruction without motion-correction (OSEM), for the gated reconstruction and for the static reference of Experiment A), in the coronal (top) and sagittal (bottom) planes

Reconstructed images for the simulated XCAT phantom are shown in Fig. 2, for the proposed MLMA and for standard reconstruction without motion-correction (OSEM), compared to the static reference *and the gated reconstruction*. Image quality metrics are reported in Table 2. MLMA significantly reduces motion-artifacts, recovering the shape of the lesion and deblurring the liver-lung interface. *MLMA outperforms the gated reconstruction*,* more significantly reducing motion-artifacts.* This is further supported by the image quality metrics, showing improved PSNR and unit SSIM. The line profiles show that MLMA preserves activity quantification.

**Table 2:** Image quality metrics for the PET activity images of the proposed MLMA, the standard reconstruction without motion-correction (OSEM), the gated reconstruction and the static reference of Experiment A)

PSNR	OSEM	MLMA	Static	*Gated*
	31.4	33.5	-	*32.5*
**SSIM**	0.78	1.0	-	*0.87*
**Variance**	4.3 × 10^− 10^	4.7 × 10^− 10^	4.8 × 10^− 10^	*4.5 × 10* ^*− 10*^

**Fig. 3:**
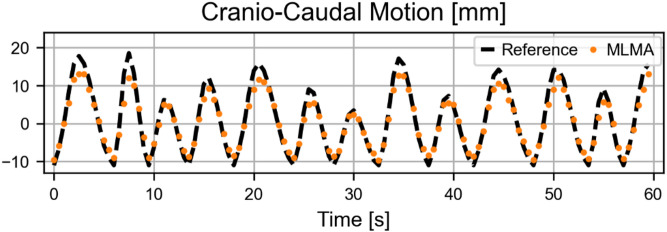
Motion profile of the lesion centroid as estimated by the proposed MLMA (dots) and programmed (dashed line) for Experiment A), in the cranio-caudal direction

The motion of the lesion is displayed in Fig. 3 for the cranio-caudal direction. The profiles show that MLMA correctly estimates the motion period but often underestimates the amplitude. This translates into RMSE of 1.0 mm, 2.2 mm and 2.2 mm for the left-right, anterior-posterior and cranio-caudal directions, respectively. The video for the time-series of estimated motion is available as Supplemental Materials.

### B Anthropomorphic Torso Phantom scan

**Fig. 4:**
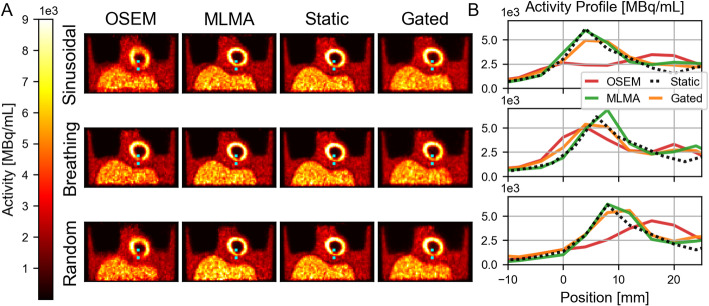
PET images (**A**) and line profiles (**B**) for the proposed MLMA, for standard reconstruction without motion-correction (OSEM) and for the static acquisitions of Experiment B)

Reconstructed images for the experimental phantom acquisitions are shown in Fig. 4, for the proposed MLMA and for reconstruction without motion-correction (OSEM), compared to the static acquisition. The image quality metrics are reported in Table 3. MLMA effectively improves image quality, recovering the shape of the heart insert and the liver-lung interface. The images from MLMA are of comparable quality to the static images, on contrast and detail but also on noise level, as all available counts are included. This is further supported by the image quality metrics, showing improved PSNR and SSIM for all the applied motions. MLMA is also observed to outperform the gated reconstructions, particularly for the acquisition with random motion.

**Table 3:** Image quality metrics for the PET activity images of the proposed MLMA, the standard reconstruction without motion-correction (OSEM), *the gated reconstruction* and the static reference of Experiment B)

	Sinusoidal				Breathing-like				Random			
	OSEM	MLMA	Static	**Gated**	OSEM	MLMA	Static	**Gated**	OSEM	MLMA	Static	**Gated**
**PSNR**	25.8	25.9	–	*26.6*	25.2	26.7	–	*25.4*	25.3	27.5	–	*26.6*
**SSIM**	0.95	0.99	–	*0.97*	0.95	0.98	–	*0.95*	0.93	0.99	–	*0.93*
**Variance**	1.8 ×10^6^	2.1 ×10^6^	2.5 ×10^6^	*2.0* *×10* ^*6*^	1.9 ×10^6^	2.1 ×10^6^	2.5 ×10^6^	*1.9* *×10* ^*6*^	1.7 ×10^6^	2.2 ×10^6^	2.5 ×10^6^	*1.7* *×10* ^*6*^

**Fig. 5:**
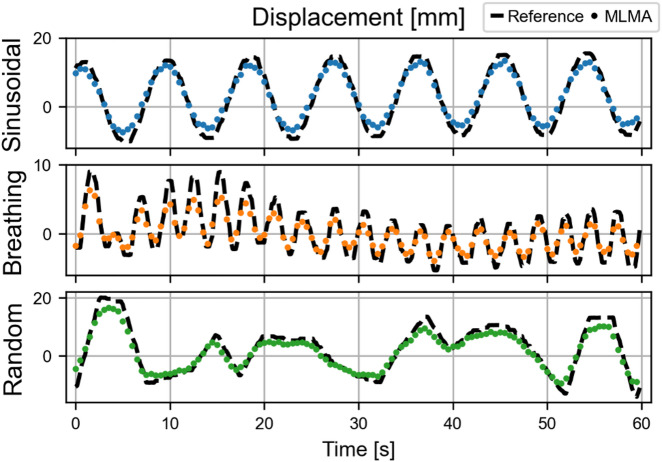
Motion profile averaged on a box surrounding the heart as estimated by the proposed MLMA (dots) and programmed (dashed line) for Experiment B), in the cranio-caudal direction

The motion profiles from MLMA are shown in Fig. 5. They show good agreement with the ground-truth, particularly on phase, with RMSE of 0.4 mm, 0.4 mm and 2.3 mm for sinusoidal motion on the left-right, anterior-posterior and cranio-caudal directions, 0.7 mm, 0.8 mm and 1.5 mm for breathing-like and 0.5 mm, 0.4 mm and 2.4 mm for random motion. Videos for the estimated motion are available as Supplemental Material.

### C Clinical patient scan

**Fig. 6:**
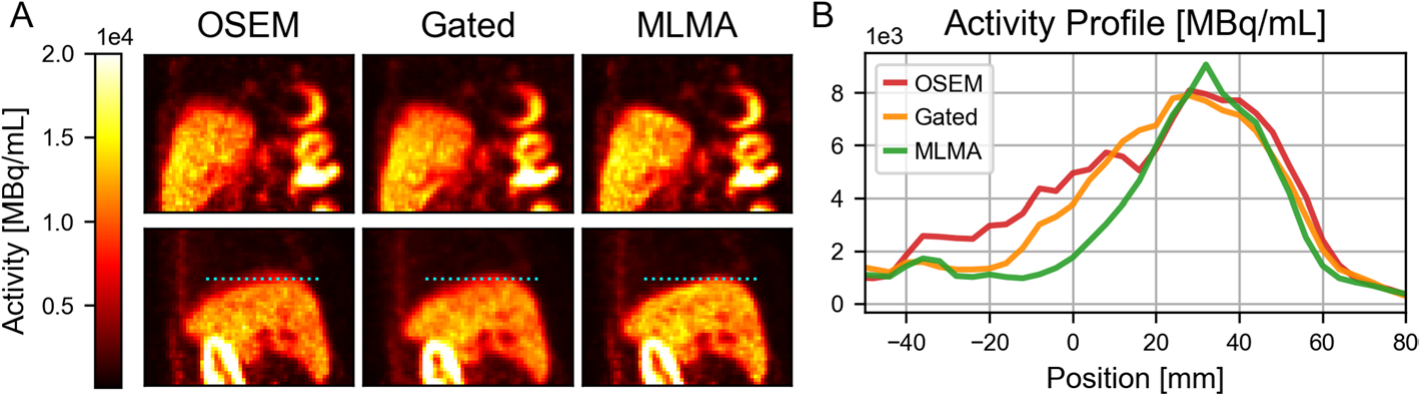
PET images A) of the proposed MLMA, of gated reconstruction and of standard reconstruction without motion-correction (OSEM) for Experiment C), in the coronal (top) and sagittal (bottom) planes alongside a line profile at the liver-lung interface B)

Reconstructed images for the clinical patient data are shown in Fig. 6, for MLMA, for gated reconstruction (all gates registered and summed) and for standard reconstruction without motion-correction (OSEM). The images from MLMA have reduced motion-artifacts, particularly at the liver-lung interface, where blurring is minimized. This effect is more pronounced than for the conventional gating approach, as highlighted by the line profile.

**Fig. 7:**
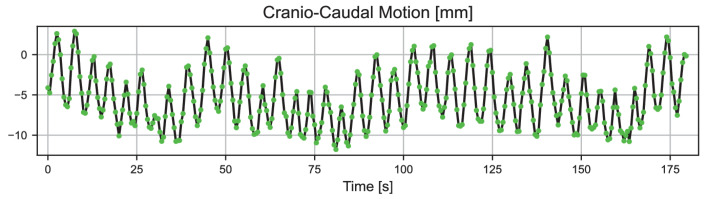
Motion profile averaged on a box at the liver-lung interface as estimated by the proposed MLMA for Experiment C), in the cranio-caudal direction

The motion profile in Fig. 7 shows varying amplitude and period in the cranio-caudal direction, as expected from free-breathing. The amplitude range of 14.7 mm and the average period of 4.5 s are realistic. The video for the time-series of estimated motion is available as Supplemental Material.

## Discussion

In this article, we have developed and demonstrated a method for motion and activity estimation at high temporal resolution, solely based on PET list mode data: MLMA. The reconstructed images show reduced or absence of motion-artifacts, with recovery of anatomical shapes and sharper interfaces. On phantom experiments, the estimated motion deviates by up to 2.5 mm on RMSE, defined as sub-voxel accuracy given the 4 mm voxel size, with the estimated activity having comparable quality to the static activity. On patient data, the estimated motion has realistic amplitude (range 14.7 mm) and period (average 4.5 s). In general, MLMA is observed to outperform conventional gated reconstruction, with the estimated activity images having less motion artifacts.

As motion is estimated solely from PET list-mode data, the motion profiles naturally combine irregular motion of varying period and amplitude. This is visible in all motion profiles and highlighted in experiment B) for the random motion, where some motion states are only observed once. In fact, it is for this acquisition with random motion that MLMA performs the best in regards to conventional gating. As such, MLMA is shown to overcome the periodicity limitation of conventional motion-correction approaches. This strength of MLMA explains the results of experiment *A) and* C), as the conventional gating method is hindered by the irregular breathing pattern, underperforming compared to MLMA. Sub-voxel accuracy is obtained for the estimated motion, with minor underestimation only for timeframes with high displacement, such as deep inhale/exhale for experiment A) and peak amplitude for B). This could be explained by an over-regularization, promoting extra smoothness.

The high temporal resolution achieved by MLMA is associated with a trade-off in computational time compared to conventional motion-correction approaches. This computational time is mostly determined by the number of timeframes, which only depends on the duration of the acquisition and the temporal resolution. However, the reported values (180 min for motion estimation and 90 min for activity estimation per alternation) could be improved by replacing the CPU-based reconstruction with a GPU-accelerated implementation. On the motion estimation step, larger voxel sizes could be used alongside data reduction (mash and span) to significantly accelerate projections.

MLMA estimates the deformation vector fields for the whole acquisition at a high temporal resolution, enabling the correction of fast motion. This is critical in many applications such as pediatric PET, where irregular and unpredictable motion degrade image quality. MLMA may enable scanning without anesthesia, by correcting the fast and irregular motion *a posteriori*. Additionally, the time-series of motion fields and the motion-corrected activity image can be combined to produce videos of the physiological motion. This may provide essential information about the physiology of anatomic structures, paving the way for future clinical applications, such as in assessing lung ventilation and heart pump function.

## Conclusion

The proposed Maximum-Likelihood Motion and Activity (MLMA) reconstruction method estimates and corrects PET acquisitions for non-rigid irregular motion. It is characterized by a high temporal resolution (2 Hz) enabled by directly processing PET list-mode data, as opposed to reconstructing short timeframes. MLMA foregoes gating, instead using all available counts to reconstruct the motion-corrected activity image and the time-series of deformation vector fields.

## Electronic Supplementary Material

Below is the link to the electronic supplementary material.

Supplementary Material 1

Supplementary Material 2

Supplementary Material 3

Supplementary Material 4

Supplementary Material 5

Supplementary Material 6

## Data Availability

The datasets generated during and/or analyzed during the current study are available from the corresponding author on reasonable request.
