# Supplementary material for: Correcting fast irregular motion in PET: maximum-likelihood motion and activity (MLMA) reconstruction
Source: EJNMMI Phys. 2026 May 26;13:77. doi: 10.1186/s40658-026-00894-0 (PMC13433875; doi:10.1186/s40658-026-00894-0)
Supplement: Supplementary file 1 [file 40658_2026_894_MOESM4_ESM.docx]

# Video1.mp4

Video 1 *Video for the simulated XCAT data of Experiment A) in the coronal (left) and sagittal (right), obtained by deforming the motion-corrected activity image with the estimated time-series of deformation vector fields as outputted by the proposed MLMA.*

# Video2.mp4

Video 2 *Video for the experimental phantom acquisitions of Experiment B) with sinusoidal (left), breathing-like (middle) and random (right) motion applied, obtained by deforming the motion-corrected activity image with the estimated time-series of deformation vector fields as outputted by the proposed MLMA.*

# Video3.mp4

Video 3 *Video for the clinical patient acquisition of Experiment C) in the coronal (left) and sagittal (right) planes, obtained by deforming the motion-corrected activity image with the estimated time-series of deformation vector fields as outputted by the proposed MLMA.*
