# Supplementary material for: Correcting fast irregular motion in PET: maximum-likelihood motion and activity (MLMA) reconstruction
Source: EJNMMI Phys. 2026 May 26;13:77. doi: 10.1186/s40658-026-00894-0 (PMC13433875; doi:10.1186/s40658-026-00894-0)
Supplement: Supplementary file 2 [file 40658_2026_894_MOESM5_ESM.docx]

Supplemental Materials

# Problem formulation

The Maximum-Likelihood Motion and Activity (MLMA) reconstruction method is built upon the motion-aware signal model for the Poisson-distributed PET data. The problem is to find an activity map $f$ and a time-series of deformation motion fields $\boldsymbol{\varphi}$ that maximize the likelihood for all measured data, from solely the positron emission tomography (PET) list-mode data $g$ and the reference attenuation map $\mu$. This is equivalent to minimize the negative log-likelihood function with regards to the activity $f$ and the motion fields $\boldsymbol{\varphi}$:

$$-L\left( f,\boldsymbol{\varphi},\mu\right)=-\sum_{t=1}^{N_{t}} L_{t}\left( f,\varphi_{t},\mu\right) \left( 1 \right)$$

$$L_{t}\left( f,\varphi_{t},\mu\right)=\sum_{i=1}^{N_{b}} \sum_{j=1}^{N_{v}} \overline{g}_{i,j}\left( f,\varphi_{t},\mu\right)-\log\left( \overline{g}_{i,j}\left( f,\varphi_{t},\mu\right) \right)*g_{t,i,j} (2)$$

where $i$ represents the line-of-response index (LOR, total $N_{b}$), $j$ is the time-of-flight bin (TOF, total $N_{v}$) ,$t$ the number of timeframes (total $N_{t}$), $g$ is the measured data and $\bar{g}$ the expected number of counts. The latter is obtained by deforming the activity map $f$ and the attenuation map $\mu$ with the motion field $\varphi_{t}$ and projecting into the LOR-space.

## Motion-free PET signal model

Let $f\left( \boldsymbol{r} \right)\in C^{+}$ and $\mu\left( \boldsymbol{r} \right)\in C^{+}$ denote the activity and attenuation maps at the spatial coordinate $\boldsymbol{r}=(x,y,z)$ respectively, where $C^{+}$denotes the set of non-negative continuous functions over $\mathbb{R}^{3}$. In the absence of motion, the measured data $g\in\mathbb{N}^{N_{b}}$ are modeled as independent Poisson distributions $g_{i,j}$ for each LOR bin $i=1,\ldots,N_{b}$ and TOF bin $j=1,\ldots,N_{v}$:

$$g_{i,j} \sim\mathrm{Poisson}\left( \overline{g}_{i,j}\left( f,\mu\right) \right) (3), \overline{g}_{i,j}=\tau a_{i}\left( \mu\right)\mathcal{H}_{i,j}f+s_{i,j} (4)$$

where $\bar{g}_{i,j}$ is the expected number of counts for LOR bin $i$ and TOF bin $j$, $\tau$ is the total duration of the acquisition and $\mathcal{H}_{i,j}f$ is the expected number of true counts per unit time along the LOR bin $i$ and TOF bin $j$ on the field-of-view (FOV) $\Omega\in\mathbb{R}^{3}$, based on the PET system response $h_{i,j}$ function for that bin:

$$\mathcal{H}_{i,j}f=\int_{\Omega} f\left( \boldsymbol{r} \right)h_{i,j}(\boldsymbol{r})d\boldsymbol{r} (5)$$

$a_{i}\left( \mu\right)$ is the attenuation factor for LOR bin $i$, calculated along the corresponding segment $L_{i}$:

$$a_{i}\left( \mu\right)=\exp\left( -\int_{L_{i}} \mu\left( \boldsymbol{r} \right)d\boldsymbol{r} \right) (6)$$

and $s_{i,j}$ is the expected number of background events (scatter and random coincidences) at LOR bin $i$ and TOF bin $j$, calculated *a posteriori* from analytical methods.

## Motion-aware PET signal model

To account for motion in time, the measured data is grouped into sequential $N_{t}$ timeframes, each of sub-second length $\Delta t$, so that $\tau=\Delta t*N_{t}$ is the full duration of the acquisition. The patient is considered static within each short timeframe $\Delta t$.

The motion at timeframe $t$ is represented by a diffeomorphism $\varphi_{t} :\mathbb{R}^{3}\to\mathbb{R}^{3}$ that deforms both the activity map $f$ and the attention map $\mu$. The deformation itself is applied through a warping operator $\mathcal{W}_{\varphi} : C^{+}\to C^{+}$ defined as:

$$\mathcal{W}_{\varphi}f = f\circ\varphi, \forall f\in C^{+} (7)$$

This way, the deformed activity and attenuation maps at timeframe $t$ are given by $\mathcal{W}_{\varphi_{t}}f$ and $\mathcal{W}_{\varphi_{t}}\mu$ respectively. By assuming that the motion is static for each timeframe $t$, the measured data $g_{t}\in\mathbb{N}^{N_{b}}$ can be modeled by a Poisson distribution for each independent entry $g_{t,i,j}$:

$$g_{t,i,j} \sim\mathrm{Poisson}\left( \overline{g}_{t,i,j}\left( f,\varphi_{t},\mu\right) \right) (8)$$

$$\overline{g}_{t,i,j}=\Delta ta_{i}\left( \mathcal{W}_{\varphi_{t}}\mu\right)\mathcal{H}_{i,j}\mathcal{W}_{\varphi_{t}}f+s_{t,i,j} (9)$$

With $s_{t,i,j}$ the number of expected background events (random and scatter coincidences) for the specific timeframe $t$.

# Optimization routine

MLMA minimizes the negative log-likelihood (see equation (1)) in an alternating way: 1) minimization with regards to the time-series of motion fields $\boldsymbol{\varphi}$ and 2) minimization with regards to the activity map $f$. The implementation scheme is reported in Table 1. The motion estimation (step 1) is based on the MOTUS framework, using projection data to directly estimate motion in the spatial domain for each short timeframe of the whole acquisition. The activity estimation (step 2) is based on standard motion-correction approaches for PET reconstruction.

| **Maximum-Likelihood Motion and Activity (MLMA) reconstruction** | | | | | |
| --- | --- | --- | --- | --- | --- |
|  | **Input*:*** *List-mode data* $g$*, attenuation map* $\mu$ | | | | |
|  | **Output:** *Motion-corrected activity map* $f$ *and time-series of deformation vector fields* $\boldsymbol{\varphi}$ | | | | |
|  | Step 0: Initialize *activity guess* $f$ by standard reconstruction (OSEM) without motion-correction (equivalent to $\boldsymbol{\varphi}$ = 0) | | | | |
|  | Initialize *motion guess* $\boldsymbol{\varphi}$ randomly | | | | |
|  | **Alternating Scheme** | | | | |
|  | 1: | **for** *each alternation N* **do** | | | |
|  | 2: |  | **Step 1** *Motion Estimation* minimizes function $\mathcal{L}$ (see equation (14)) with regards to $\boldsymbol{\varphi}$ | | |
|  |  |  | $\varphi_{t}^{*}=\underset{\varphi_{t}}{arg min} \mathcal{L}_{t}\left( f,\varphi_{t},\mu\right) (10)$ | | |
|  | 3: |  |  | **for** each timeframe *t* **repeat** *M* **times** | |
|  | 4: |  |  |  | Deform *activity guess* $f$ with *motion guess* $\varphi_{t}$  $f_{t}=\mathcal{W}_{\varphi_{t}}f (11)$ |
|  | 5: |  |  |  | Deform *attenuation map* $\mu$ with *motion guess* $\varphi_{t}$  $\mu_{t}=\mathcal{W}_{\varphi_{t}}\mu(12)$ |
|  | 6: |  |  |  | Calculate *expected number of counts* $\overline{g}_{t}$  $\overline{g}_{t,i}=\Delta ta_{i}\left( \mu_{t} \right)\mathcal{H}_{i}f_{t}+s_{t,i} (13)$ |
|  | 7: |  |  |  | Calculate loss $\mathcal{L}$ from *expected* $\overline{g}_{t}$ and *measured counts* $g_{t}$  $\mathcal{L}_{t}=-L_{t}+\alpha\mathcal{R}_{t}+\beta\mathcal{T}_{t}+\eta\mathcal{C}_{t} =\sum_{i=1}^{N_{b}} \overline{g}_{t,i}-\log\left( \overline{g}_{t,i} \right)*g_{t,i} +\alpha\sum_{p\in\left\{ x,y,z \right\}} \int_{\Omega} \left\vert{\Delta\varphi}_{t}^{p}\left( \boldsymbol{r} \right) \right\vert^{2}d\boldsymbol{r}+\beta\sum_{p\in\left\{ x,y,z \right\}} \int_{\Omega} \left\vert\varphi_{t-1}^{p}\left( \boldsymbol{r} \right)- \varphi_{t}^{p}\left( \boldsymbol{r} \right) \right\vert^{2}d\boldsymbol{r}+\eta\sum_{p\in\left\{ x,y,z \right\}} \int_{\Omega^{*}} \left\vert1-\det\left( J\left( \varphi_{t}^{p}\left( \boldsymbol{r} \right) \right) \right) \right\vert^{2}d\boldsymbol{r} (14)$ |
|  | 8: |  |  |  | Update *motion guess* $\varphi_{t}$ through the *NAdam* optimizer  $\varphi_{t}^{*}=\underset{\varphi_{t}}{\mathrm{NAdam}} \left( \mathcal{L}_{t} \right) (15)$ |
|  | 9: |  |  |  | |
|  | 10: |  | **Step 2** Activity Estimation minimizes function $-L$ (see equation (1)) with regards to $f$  $f^{*}=\underset{f}{arg min} L\left( f,\boldsymbol{\varphi},\mu\right) (16)$ | | |
|  | 11: |  |  | **for** each iteration *I* and subset *S* **repeat for** all timeframes *t* **in** subset *S* | |
|  | 12: |  |  |  | Deform *activity guess* $f$ with *motion guess* $\varphi_{t}$  $f_{t}=\mathcal{W}_{\varphi_{t}}f (17)$ |
|  | 13: |  |  |  | Deform *attenuation map* $\mu$ with *motion guess* $\varphi_{t}$  $\mu_{t}=\mathcal{W}_{\varphi_{t}}\mu(18)$ |
|  | 14: |  |  |  | Calculate *expected number of counts* $\overline{g}_{t}$  $\overline{g}_{t,i,j}=\Delta ta_{i}\left( \mu_{t} \right)\mathcal{H}_{i,j}f_{t}+s_{t,i,j} (19)$ |
|  | 15: |  |  |  | Calculate update from *expected* $\overline{g}_{t}$ and *measured counts* $g_{t}$  $f_{t}^{*}=\underset{f}{arg min} L_{t}\left( f,\boldsymbol{\varphi},\mu\right)=\underset{f}{arg min} \left( \sum_{i=1}^{N_{b}} \sum_{j=1}^{N_{v}} \overline{g}_{t,i,j}-\log\left( \overline{g}_{t,i,j} \right)*g_{t,i,j} \right) (20)$ |
|  | 16: |  |  |  | Undeform *updated* *activity* $f_{t}^{*}$ with *inverse* *motion guess* $\varphi_{t}^{-1}$  $f=\mathcal{W}_{\varphi_{t}^{-1}}f_{t}^{*} (21)$ |

Table 1 Pseudocode for the Maximum-Likelihood Motion and Activity (MLMA) reconstruction method.

## Motion Model

MLMA uses a spatially-smooth motion model, parameterized by lower-dimension 3D cubic B-spline basis functions [1]. For each timeframe $t$, the motion $\varphi_{t}$ is modeled by a set of coefficients $\boldsymbol{\theta}_{t}$ applied to $N_{\theta}$ uniformly-spaced control points and evaluated at every point in space $\boldsymbol{r}$:

$$\varphi_{t}\left( \boldsymbol{r},\boldsymbol{\theta}_{t}^{x},\boldsymbol{\theta}_{t}^{y},\boldsymbol{\theta}_{t}^{z} \right)=\boldsymbol{r}+\left( \begin{matrix} \boldsymbol{b}^{x}\left( \boldsymbol{r} \right)*\boldsymbol{\theta}_{t}^{x} \\ \boldsymbol{b}^{y}\left( \boldsymbol{r} \right)*\boldsymbol{\theta}_{t}^{y} \\ \boldsymbol{b}^{z}\left( \boldsymbol{r} \right)*\boldsymbol{\theta}_{t}^{z} \end{matrix} \right) (22)$$

where $\boldsymbol{b}^{p}\left( \boldsymbol{r} \right)\in\mathbb{R}^{1\times N_{\theta}^{p}}$ are the row-vectors with $N_{\theta}^{p}$ 3D-Bspline basis functions, evaluated at the coordinate $\boldsymbol{r}$, and $\boldsymbol{\theta}_{t}^{p}\in\mathbb{R}^{N_{\theta}^{p}\times1}$ denotes the expansion coefficients. The 3D basis functions are constructed as a Kronecker product of three 1D bases with a spline order (i.e. the number of control points) of $\kappa$ each. If the same basis is used for all the three components of the motion-field, the total number of coefficients for the spline model is $N_{\theta}=\sum_{p} N_{\theta}^{p}=3\kappa^{3}$. As such, the number of coefficients is significantly reduced, making the motion-estimation problem solvable for highly-undersampled data, corresponding to short very-low-count projection data.

## Regularization

To further improve convergence speed and motion accuracy, *a priori* knowledge can be added to the objective function. The spatial smoothness assumption can be further enforced by a regularization term $\mathcal{R}_{t}$, adding the Laplace operator $\Delta$ of the deformation vector fields $\varphi_{t}$ to the objective function:

$$\mathcal{R}_{t}\left( \varphi_{t} \right):=\sum_{p\in\left\{ x,y,z \right\}} \int_{\Omega} \left| {\Delta\varphi}_{t}^{p}\left( \boldsymbol{r} \right) \right|^{2}d\boldsymbol{r} (23)$$

Additionally, motion is expected to be continuous and smooth between consecutive frames and no motion is expected outside the subject. This translates into two extra regularization terms: a temporal smoothness term $\mathcal{T}_{t}$ regularizing the square deviation of the deformation vector fields $\varphi_{t}$ between two consecutive timeframes and a spatial compressibility term $\mathcal{C}_{t}$ regularizing compressibility in regions outside of the subject, through the determinant of the Jacobian of $\varphi_{t}$:

$$\mathcal{T}_{t}\left( \boldsymbol{\varphi}_{t} \right):=\sum_{p\in\left\{ x,y,z \right\}} \int_{\Omega} \left| \varphi_{t-1}^{p}\left( \boldsymbol{r} \right)- \varphi_{t}^{p}\left( \boldsymbol{r} \right) \right|^{2}d\boldsymbol{r} \left( 24 \right),$$

$$\mathcal{C}_{t}\left( \boldsymbol{\varphi}_{t} \right):=\sum_{p\in\left\{ x,y,z \right\}} \int_{\Omega^{*}} \left| 1-\det\left( J\left( \varphi_{t}^{p}\left( \boldsymbol{r} \right) \right) \right) \right|^{2}d\boldsymbol{r} (25)$$

These regularization terms are weighted by $\alpha$, $\beta$ and $\eta$ respectively, and added to the negative log-likelihood function. As such, the objective function for the motion-estimation problem of each timeframe $t$ takes the form:

$$\mathcal{L}_{t}:=-L_{t}+\alpha\mathcal{R}_{t}+\beta\mathcal{T}_{t}+\eta\mathcal{C}_{t} (26)$$

As TOF is disabled on the motion estimation step, in order to meet the memory constraints associated with GPU acceleration, the log-likelihood function $L_{t}$ takes the form:

$$L_{t}\left( f,\varphi_{t},\mu\right)=\sum_{i=1}^{N_{b}} \overline{g}_{i}\left( f,\varphi_{t},\mu\right)-\log\left( \overline{g}_{i}\left( f,\varphi_{t},\mu\right) \right)*g_{t,i} (27), g_{t,i}=\sum_{j=1}^{N_{v}} g_{t,i,j} (28)$$

## Parameter configuration

MLMA was configured with the values in Table 2. For motion estimation, the NAdam optimizer [2] was used with reference values for 10 iterations *M*. The initial guess was randomly initialized for the first timeframe, with subsequent timeframes using the previous solution for initialization. For the activity estimation step, the values used (3 iterations *I* for 21 subsets *S*) correspond to reference values provided by the vendor.

|  | Dataset | NAdam update step | Number of B-spline coefficients per direction $\kappa$  *- equation (22) -* | Spatial smoothness $\mathcal{R}_{t}$ weight $\alpha$  *- equation (23) -* | Temporal smoothness $\mathcal{T}_{t}$ weight $\beta$  *- equation (24) -* | Compressibility $\mathcal{C}_{t}$ weight $\eta$  *- equation (25) -* |
| --- | --- | --- | --- | --- | --- | --- |
| A | XCAT phantom | 0.4 | 10 | $5\times{10}^{-8}$ | $1\times{10}^{-9}$ | $1\times{10}^{-1}$ |
| B | Moving Phantom |  | 5 | $4\times{10}^{-8}$ | $4\times{10}^{-8}$ | $4\times{10}^{-1}$ |
| C | Patient |  | 10 | $4\times{10}^{-8}$ | $4\times{10}^{-8}$ | $4$ |

Table 2 Summary of the parameters used for MLMA.

For the B-splines of the motion model, the number of coefficients is recommended to be the smallest possible that still accurately models the motion. This is to prevent the optimizer from fitting motion to the inherent noise of the short very-low-count timeframes of projection data. We empirically chose the values in Table 2.

As for the optimization parameters, it is recommended to start by tuning the step-size parameters of the optimizer for a small set of timeframes, without regularization. The rationale is that this parameter has a direct effect on the convergence speed. On tuning the regularizers, it is recommended to start with the compressibility term $\mathcal{C}$, followed by the spatial-smoothness term $\mathcal{R}$ and the temporal-smoothness term $\mathcal{T}$.The values were chosen empirically, from visual inspection of the deformation vector fields.

# Deformation vector fields for clinical patient data

Activity images for the clinical patient data are shown in Figure SM1, for the proposed MLMA at specific timeframes. The corresponding deformation vector fields are overlapped, alongside the corresponding motion profile in time.

Figure SM1 Motion profile A) and PET images B) from MLMA at specific timeframes in the coronal (top) and sagittal (bottom) planes, overlapped with the corresponding deformation vector fields.

# References

1.	Rueckert D, Sonoda LI, Hayes C, Hill DL, Leach MO, Hawkes DJ. Nonrigid registration using free-form deformations: application to breast MR images. IEEE Trans Med Imaging. 1999;18:712-21. doi:10.1109/42.796284.

2.	Dozat T. Incorporating Nesterov Momentum into Adam. Proceedings of the 4th International Conference on Learning Representations. San Juan; 2016.
