# Supplementary material for: Correcting fast irregular motion in PET: maximum-likelihood motion and activity (MLMA) reconstruction
Source: EJNMMI Phys. 2026 May 26;13:77. doi: 10.1186/s40658-026-00894-0 (PMC13433875; doi:10.1186/s40658-026-00894-0)
Supplement: Supplementary file 3 [file 40658_2026_894_MOESM6_ESM.docx]

**Reviewer reports**
**Reviewer #1:**

The revision has addressed the reviewer comments. I have only a few minor comments.

> We thank you for the remarks.

**SUPPLEMENTAL MATERIALS**

There are problems with the numbering of the equations. Some equations are numbered, some are not, and the first one that is numbered has label (2). Most of the equations in table 1 are numbered, but their numbers seem random and they are not unique (5 of the equations have label (2)).

> We have updated the numbering of equations to improve readability. All equations follow unique numbering throughout the Supplemental Materials.

Just before the second equation (bottom of page 1), it is stated that the data are modeled as independent Poisson distributions g_{i,t}. I think that should be g_{i,j}, as in that equation.

> We thank you for your comment. It has been corrected.

On page 2 of the supplemental materials, it is stated that the background events (scatter and random coincidences) are "calculated a posteriori from analytical methods". "A posteriori" suggests that the motion correction was done without correcting for background events, and that a final reconstruction is made after the motion estimation, possibly even accounting for motion effects on the scatter estimates. However, in the paper (line 145), it is stated that "clinical corrections for scatters and randoms estimated by the scanner were applied". This suggests that the effect of motion on randoms and scatters was ignored, and that the background obtained from the static scan was used for all frames (accounting for frame duration). Could you explain a bit better in the paper and the supplemental materials how and when exactly the background events were accounted for?

> Yes, we did not account for the effects of motion in the randoms and scatters as we hypothesized that these are only minimally affected by motion. These were estimated in the initialization step of the proposed method, namely the first activity estimation without motion correction. Randoms and scatters are part of the signal model, as described in the Supplemental Materials (equation 4). We have made this more clear in the manuscript: “The expected number of counts $\bar{g}$ takes into account the effects of normalization, randoms and scatters directly in the signal model. These contributions are estimated in the initialization step of MLMA, using the motion-blurred image to estimate the time-averaged correction factors.” (page 6, lines 85-88)

In table 2, there is a column labeled "B-spline coefficients \theta_t - equation (4) -". I think it probably gives \kappa, the number of coefficients along one dimension, which is discussed after equation (4).

> We have modified table 2 to explicitly mention the number of coefficients per direction $\kappa$ instead of the coefficients themselves $\theta_{t}$. So it now reads: “Number of B-spline coefficients per direction $\kappa$ *- equation (22)*” (Supplemental Materials, page 7, table 2)

The image has 128 x 128 x 54 voxels with isotropic size. If I understood correctly, then the same number of control points is used in all three dimensions. That suggests that in the axial dimension (54 voxels), the motion is more flexible than in the other two dimensions. Is that just a side effect of using the most convenient approach, or does this improve things for some reason?

> That is correct, we do use the same number of control points in all dimensions for practicality of implementation, which translates into more flexibility in the axial dimension.

THE PAPER

The first word of the abstract should be "positron", not "position".

> Thank you. It has been corrected in the manuscript.

**Reviewer #2:**

The authors have revised the manuscript in response to the previous review. The manuscript has partially improved by including additional results and gaining in clarity. […] However, some comments remain to be addressed before the manuscript can be considered for publication.

> We thank you for the remarks.

**Major revisions**
The authors were asked to compare their proposed approach with other motion compensation methods. They have partially addressed this comment by adding a gated reconstruction for the clinical patient data. Please consider extending this comparison to the other experiments, i.e., the XCAT phantom simulations and the anthropomorphic phantom scan. This would allow a more comprehensive comparison of reconstruction quality and duration, highlighting strengths and potential weaknesses of the proposed MLMA.

> Thank you for the suggestion. We have complemented the analysis of the other two experiments by including the suggested gated reconstructions. As such, figure 2 and table 2 (page 13) as well as figure 4 and table 3 (page 15) have been updated. We also added the following discussion points: “In general, MLMA is observed to outperform conventional gated reconstruction, with the estimated activity images having less motion artifacts.” (page 17, line 261-263) and “In fact, it is for this acquisition with random motion that MLMA performs the best in regards to conventional gating. As such, MLMA is shown to overcome the periodicity limitation of conventional motion-correction approaches.” (page 17, lines 267-269)


**Minor revisions**

Please consider reporting the complete reconstruction time for each experiment, for both MLMA and the gated-based method.

> The reconstruction time for MLMA is the same for all the experiments, as it is determined by the acquisition duration and temporal frequency. We also observed the same reconstruction time for all the gated-based reconstructions, which is determined by the number of gates.

> We have updated the manuscript to clarify on this point, adding the reconstruction time for the gated-based method: “The reconstruction time of MLMA was the same for all experiment, clocking at 9 hours. Per alternation, the motion estimation step took 180 minutes and the activity estimation step took 90 minutes. Reconstruction time for the gated-reconstruction was 15 minutes, including the overhead to sort the gates. For the clinical patient data, an additional 20 minutes were required to obtain the gating signal from the list-mode data.” (page 11, lines 187-191) and “This computational time is mostly determined by the number of timeframes, which only depends on the duration of the acquisition and the temporal resolution.” (page 18, lines 276-278)

- (Very minor) The authors state that the reconstructed volume is $128 \times 128 \times 54$ with isotropic voxels. However, on the plotted images (figures 2A, 4A, and 6A) the vertical dimension (axial) seems greater than stated (it appears to be approximately 80 voxels).

> This is a good observation. We confirm that the reconstructed volume is $128\times128\times54$ with isotropic voxels. The plotted images may suggest a different size because we have cropped them in the horizontal dimension for visualization purposes.
